# Prevalence and Correlates of Low Resilience: Aftermath of the Fort McMurray Wildfire Disaster

**DOI:** 10.1192/j.eurpsy.2023.645

**Published:** 2023-07-19

**Authors:** M. K. Adu, E. Eboreime, R. Shalaby, A. Sapara, B. Agyapong, G. Obuobi-Donkor, W. Mao, E. Owusu, F. Oluwasina, H. Pazderka, V. Agyapong

**Affiliations:** ^1^Psychiatry, Dalhousie University, Halifax NS, Halifax; ^2^Psychiatry, University of Alberta, Edmonton, Canada

## Abstract

**Introduction:**

The Fort McMurray wildfire (2016) was one of the most expensive and devastating natural disasters that ever happened in the history of Canada. According to the Insurance Bureau of Canada (2016), the cost of this disaster was estimated at USD 3.6 billion in insured losses. Despite the fundamental role of resilience in the daily functioning of individuals in the form of a protective shield that ameliorates the devastating impact of disasters on their mental well-being, to date, the long-term impact of wildfires on resilience and its associated predictors of low resilience has not been well studied and evaluated.

**Objectives:**

The study aimed to enhance the understanding of the psychological impact of wildfires through the evaluation of the prevalence and predictors of resilience among the affected residents of Fort McMurray five years after the devastating wildfires.

**Methods:**

This study applied a cross-sectional survey design which was used to gather quantitative data through an online-based self-administered questionnaire. The surveys included standardized rating scales for resilience (BRS), depression (PHQ-9), anxiety disorder (GAD-7), and post-traumatic stress disorder (PTSD) (PCL-C) was used to measure the prevalence of resilience and its demographic, clinical, as well as wildfire-related predictors. The data collection spanned between April and June of 2021. Data were analyzed using the Statistical Package for Social Sciences (SPSS) version 25 and univariate analysis with done using a chi-squared test and binary logistic regression analysis.

**Results:**

A total of 249 residents accessed the online survey and 186 completed the survey. Therefore, there was a response rate of 74.7%. Most of the respondents were females (85.5%, 159), above 40 years of age (81.6%, 80), employed (94.1%, 175), and in a relationship (71%, 132). The study identified two variables, thus having PTSD symptoms (OR = 2.85; 95% CI: 1.06–7.63), and the age of respondents significantly predicted low resilience in our sample. The prevalence of low resilience in our sample was found to be about 37.4%.

**Conclusions:**

The study finding demonstrated that age and the presence of PTSD were the independent significant risk factors associated with low resilience in the affected population of Fort McMurray five years after the devastating wildfire disaster. This result further provides new information about the association between resilience, demographic, and clinical characteristics while adding to the rising body of evidence on the benefits of resilience in individuals during and after disasters. However, further research is needed to enhance understanding of the pathways to

**Disclosure of Interest:**

None Declared

